# Novel BMI cutoff points for obesity diagnosis in older Hispanic adults

**DOI:** 10.1038/s41598-024-65553-9

**Published:** 2024-11-11

**Authors:** Natalia Esparza-Hurtado, Alexandro J. Martagon, Delia Patricia Hart-Vazquez, Alejandra Rodríguez-Tadeo, Rogelio González-Arellanes

**Affiliations:** 1 Escuela de Medicina y Ciencias de la Salud, Tecnologico de Monterrey, 64700 Mexico City, Mexico; 2 Institute for Obesity Research, Tecnologico de Monterrey, 64700 Mexico City, Mexico; 3 Unidad de Investigación de Enfermedades Metabólicas, Instituto Nacional de Ciencias Médicas y Nutrición Salvador Zubirán, 14080 Mexico City, Mexico; 4 Instituto de Ciencias Biomédicas, Departamento de Ciencias de la Salud, Universidad Autónoma de Ciudad Juárez, 32310 Juarez City, Chihuahua Mexico

**Keywords:** Obesity, Older adults, BMI, Body composition, Diseases, Medical research

## Abstract

Current body mass index (BMI) cutoff points (≥ 30 kg/m^2^) underestimate obesity prevalence in older adults. The aim of the present study was to propose new BMI cutoff points for identifying obesity in older Hispanic adults. In this study, new internally derived (ID) BMI cutoff point for obesity in older Hispanic adults was developed by analyzing data from the National Health and Nutrition Survey of 2018–2019 from Mexico. To evaluate the performance/validation of this newly proposed cutoff point, data from the "Study of the 1000", conducted in Northern Mexico, was utilized. Sensitivity, and negative predictive value (NPV) were assessed using receiver operating characteristic analysis, with obesity defined by fat mass index (FMI; ≥ 9.0 kg/m^2^ for men and ≥ 12.0 kg/m^2^ for women) as the reference method. The newly proposed ID BMI cutoff point was ≥ 27.2 kg/m^2^ which demonstrated high sensitivity (≥ 99.4%) and NPV (≥ 99.5%) in the total sample. Furthermore, the prevalence of obesity estimated by the new BMI cutoff point was comparable to that estimated by the FMI. The newly proposed BMI cutoff point provides a more accurate identification of obesity in older Hispanic adults. These findings have implications for improving obesity diagnosis and management in this population.

## Introduction

Obesity poses significant global public health challenges, particularly among older adults who are highly susceptible to this condition. In recent decades, there has been a significant increase in the elderly population^1^, which has been accompanied by a parallel rise in the prevalence of obesity. Approximately 15% of older men and 18% of older women worldwide are living with obesity^2^. In developing countries like Mexico, the prevalence of obesity is 34% in older adults^3^. In this age group, there is a documented tendency for fat accumulation and redistribution to the abdominal region, attributed to metabolic and hormonal changes^4^. Obesity in this population holds significant importance due to the heightened risk it poses to the overall health of older adults. For instance, it substantially increases the likelihood of developing type II diabetes, and cardiovascular diseases^5^. Consequently, there is a pressing need for a timely diagnosis of obesity in older adults.

According to the World Health Organization, a body mass index (BMI) ≥ 30 kg/m^2^ is the recommended cutoff point for diagnosing obesity in adults of both sexes. However, BMI has several limitations in accurately diagnosing obesity. This is because the BMI is derived from total body weight and does not differentiate between the weight contributed by fat and fat-free mass^6^. Certainly, Aleman-Mateo et al.^7^ found in their study that, in both sexes, older Mexican adults exhibited a higher level of body fat compared to their older Caucasian and African American counterparts, despite having the same BMI. In a study by Romero-Corral et al.^8^, it was found that the sensitivity of BMI (≥ 30 kg/m^2^) to identify obesity in adults (20 to 79.9 years) was 36% in men and 49% in women when compared to body fat percentage. This suggests that BMI significantly underestimated the prevalence of obesity by approximately 50% when compared to body fat percentage (%BF) (obesity; > 25%BF for men and > 35%BF for women). However, it is important to note that %BF is not a comprehensive indicator of obesity as it does not consider the individual's height.

Hence, alternative indicators have been explored to assess obesity more accurately. One such method is the fat mass index (FMI), which correlates fat mass with height^9^. In 2024, Alemán-Mateo et al.^10^ developed cutoff points for obesity based on FMI in older Latin American adults. However, utilizing FMI requires estimating fat mass through body composition techniques, which often necessitates expensive equipment and may not always be readily available in clinical or epidemiological settings. The primary advantage of using BMI lies in its practicality and broad applicability in clinical and epidemiological settings. However, it is crucial to establish reliable BMI cutoff points for identifying obesity in older adults that account for factors such as ethnicity and sex. In this sense, the aim of the present study is to propose new BMI cutoff points for obesity in older Hispanic adults.

## Methods

### Database

The present study conducted a sub-analysis using data from two cross-sectional studies. The new internally derived (ID) BMI cutoff points for obesity in older Mexican adults were determined based on a nationally representative sample from the "National Health and Nutrition Survey" (ENSANUT, 2018–2019). The methodology details can be found in Shamah-Levy et al.^11^. To evaluate the performance of the new ID BMI cutoff point, data from the "Study of the 1000" (EDLM) conducted in 2009–2010 in Northern Mexico (Chihuahua, Mexico) by the Universidad Autónoma de Ciudad Juárez (Ethics committee approval number UACJ-02-2009) was used. The inclusion criteria for both databases were subjects aged 60 to 90 years old with a BMI range of 18.5 to 42.5 kg/m^2^ and without short stature (less than 1.50 m in women and 1.60 m in men). Exclusion criteria encompassed subjects outside the age range of 60 to 90 years, with a BMI < 18.5 or > 42.5 kg/m^2^, with short stature, or with missing values. The variables obtained from both databases were age, sex, height, and weight. BMI was calculated dividing weight in kilograms by height in meters squared.

### Obesity classification

FMI was employed as a reference indicator for obesity, utilizing the cutoff points published by Alemán-Mateo et al.^10^. These cutoff points, based on data from the cross-sectional study called “Improving the Quality of life of Older People Through Early Diagnosis of Sarcopenia”, identified FMI thresholds of ≥ 9.0 kg/m^2^ for men and ≥ 12.0 kg/m^2^ for women. It is noteworthy that these cutoff points correspond to the last two quintiles of the distribution of FMI for older adults from ten Latin American countries (Argentina, Brazil, Chile, Costa Rica, Guatemala, Honduras, Mexico, Peru, Paraguay, and Uruguay).

The FMI was calculated dividing the FM in kilograms by height in meters squared. Particularly, fat mass was estimated by the following predictive equation designed and validated in older Hispanic adults using the 4-compartment model as a reference method^12^:

$${\text{FM }} = \, \left( {0.{4}0{9 }*{\text{ weight}}} \right) \, - \, \left( {{8}.0{63 }*{\text{ sex}}} \right) \, + \, \left( {0.{549 }*{\text{ BMI}}} \right) \, - { 12}.{899}.$$

where weight in kilograms, women = 0 and men = 1, and BMI in kg/m^2^.

### Statistical analyses

The statistical analysis was performed in STATA version 13.1. The distribution data was tested by the Skewness test and histogram plot. Sex differences were probed by a two-sample independent t-test. Sensibility, specificity, negative predictive value, and positive predictive value for obesity using a BMI of ≥ 30 kg/m^2^ were determined. Those cutoff points -by sex- with a high (≥ 95%) value of sensibility, specificity, negative predictive value (NPV), positive predictive value (PPV), likelihood ratio positive (LR+; > 10), and low (< 1.0) likelihood ratio negative (LR-) were selected as the new ID BMI cutoff points for obesity. Afterwards, the performance of new ID BMI cutoff point (sensibility, specificity, NPV, and PPV) was assessed in the EDLM database. It is important to note that obesity by FMI kg/m^2^ was considered as the reference indicator.

### Ethics approval and consent to participate

This study was conducted according to the guidelines laid down in the Helsinki Declaration, and all procedures involving human subjects were approved by the Ethics Committee of the UACJ (UACJ-02-2009). Informed written consent was obtained from all subjects.

## Results

The analysis of the ENSANUT 2018–2019 dataset, following the application of inclusion and exclusion criteria, involved 1630 older adults (47.4% women) aged between 60 and 90 years. The participants had a BMI ranging from 18.5 to 42.6 kg/m^2^ and an FMI ranging from 3.6 to 21.8 kg/m^2^. In the EDLM analysis, data from 1,055 older adults (69.3% women) aged between 60 and 90 years were utilized. The BMI range in this dataset was 20.1 to 42.3 kg/m^2^, and the FMI range was 4.6 to 21.6 kg/m^2^. Comparative analysis revealed that, in both databases, older men had significantly higher mean values of age, height, and weight compared to older women. However, older women exhibited higher mean values of BMI, fat mass, and FMI compared to older men (Table 1, p < 0.05).

**Table 1:** General characteristics of subjects included in the present study.

ENSANUT 2018–2019 study	Women (*n* = 772)	Men (*n* = 858)	Total (*n* = 1630)
Age, years	68.0 ± 6.5	69.0 ± 6.9*	68.6 ± 6.7
Weight, kg	70.4 ± 12.0	77.3 ± 13.3*	74.0 ± 13.1
Height, cm	155.4 ± 0.05	167.2 ± 0.05*	161.6 ± 0.07
BMI, kg/m^2^	29.1 ± 4.8*	27.6 ± 4.4	28.3 ± 4.6
Fat mass, kg	31.9 ± 7.4*	25.8 ± 7.7	28.7 ± 8.2
Fat-free mass, kg	38.5 ± 4.7	51.5 ± 5.7*	45.3 ± 8.3
FMI, kg/m^2^	13.2 ± 3.0*	9.2 ± 2.6	11.1 ± 3.5

EDLM study	Women (*n* = 731)	Men (*n* = 324)	Total (*n* = 1055)
Age, years	71.2 ± 6.8	73.4 ± 6.8*	71.9 ± 6.9
Weight, kg	67.9 ± 11.7	72.8 ± 10.6*	69.5 ± 11.6
Height, cm	157.5 ± 0.05	167.5 ± 0.05*	160.6 ± 0.07
BMI, kg/m^2^	27.4 ± 4.4*	25.9 ± 3.5	26.9 ± 4.2
Fat mass, kg	29.9 ± 7.0*	23.1 ± 6.2	27.8 ± 7.5
Fat-free mass, kg	38.0 ± 4.8	49.8 ± 4.6*	41.6 ± 7.2
FMI, kg/m^2^	12.1 ± 2.8*	8.2 ± 2.1	10.9 ± 3.1

*FMI* fat mass index, *BMI* body mass index. The sex comparison was tested by a two-sample independent t-test, *p ≤ 0.05.

### Performance of BMI cutoff point of ≥ 30 kg/m^2^ to classify obesity.

The prevalence of obesity in older adults was underestimated when using a BMI cutoff point of ≥ 30 kg/m^2^ in comparison with the prevalence estimated by FMI in ENSANUT 2018–2019 study. This underestimation of obesity prevalence was ~ 40% in the total sample, ~ 37% in older women, and ~ 44% in older men. Significantly, there were notable differences in the confidence intervals of obesity prevalence when comparing FMI and BMI measurements. This suggests that the two indicators can provide estimates of obesity that have different variability and precision. Additionally, the sensitivity of BMI was determined to be 59.8% in the total sample, with a NPV of 64.6%. For older women, the sensitivity and NPV of BMI were 63.1% and 60.5%, respectively. In the case of older men, the sensitivity of BMI was 56.3%, with an NPV of 67.7% (Table 2).

**Table 2:** Performance of BMI of ≥ 30 kg/m^2^ to classify obesity in older adults from ENSANUT 2018–2019 data.

	Prevalence of obesity, (CI)	Sensitivity	Specificity	NPV	PPV
Total sample					
FMI, ref	57.7%, (55.3–60.1)	–	–	–	–
BMI	34.5%, (32.2–36.9)	59.8%	100%	64.6%	100%
Women					
FMI, ref	63.9%, (60.4–67.3)	–	–	–	–
BMI	40.3%, (36.8–43.8)	63.1%	100%	60.5%	100%
Men					
FMI, ref	52.2%, (48.8–55.6)	–	–	–	–
BMI	29.4%, (26.3–32.5)	56.3%	100%	67.7%	100%

*FMI* fat mass index, *BMI* body mass index, *CI* Confidence intervals, *NPV* Negative predictive value, *PPV* Positive predictive value.

### The new internally derived BMI cutoff point to classify obesity

According to the ROC analysis, it was determined that the new BMI cutoff point proposed to classify individuals with obesity in the total sample and in both sexes is ≥ 27.2 kg/m^2^. This cutoff point exhibited a sensitivity and specificity value of 98.5% and 98.3%, respectively, in the total sample (Table 3).

**Table 3:** ROC analysis of BMI to classify obesity in older adults by sex from ENSANUT 2018–2019 data.

BMI value	Sensitivity	Specificity	LR+	LR−
Women				
≥ 27.1	100.0%	97.9%	46.500	0.000
** ≥ 27.2**	**99.8%**	**99.3%**	**139.216**	**0.002**
≥ 27.3	98.6%	99.3%	137.518	0.014
Men				
≥ 27.1	99.1%	96.6%	29.024	0.009
** ≥ 27.2**	**97.1%**	**97.6%**	**39.810**	**0.029**
≥ 27.3	96.7%	98.5%	66.045	0.034
Total				
≥ 27.1	99.6%	97.1%	34.303	0.004
** ≥ 27.2**	**98.5%**	**98.3%**	**56.562**	**0.015**
≥ 27.3	97.7%	98.8%	84.111	0.023

Significant values are in bold.

*BMI* body mass index, *LR+* Likelihood ratio positive, *LR−* Likelihood ratio negative.

### Performance of the new ID BMI cutoff point to classify obesity

To evaluate the effectiveness of the newly proposed ID BMI cutoff point for identifying obesity in older adults, we calculated the sensitivity, specificity, NPV, and PPV using the EDLM database. Remarkably, the new ID BMI cutoff point demonstrated consistently high sensitivity (≥ 98.0%) and NPV (≥ 99.0%) in both sexes. Furthermore, there were no significant differences in the confidence intervals for obesity prevalence between FMI and new ID BMI measurements (Table 4).

**Table 4:** Evaluation of the new ID BMI cutoff points of ≥ 27.2 kg/m^2^ to classify obesity in older adults from EDLM database.

	Prevalence of obesity, (CI)	Sensitivity	Specificity	NPV	PPV
Women					
FMI, ref	47.2% (43.5–50.9)	–	–	–	–
New ID BMI	47.3% (43.7–51.0)	99.7%	99.5%	99.7%	99.4%
Men					
FMI, ref	34.3% (29.1–39.7)	–	–	–	–
New ID BMI	34.9% (29.7–40.3)	98.2%	98.1%	99.1%	96.5%
Total					
FMI, ref	43.2% (40.2–46.3)	–	–	–	–
New ID BMI	43.5% (40.5–46.6)	99.4%	99.0%	99.5%	98.7%

*FMI* fat mass index, *BMI* body mass index, *CI* Confidence intervals, *NPV* Negative predictive value, *PPV* Positive predictive value.

## Discussion

In the case of older Mexican adults, the Official Mexican Standard for the comprehensive treatment of overweight and obesity (NOM-008-SSA3-2017) established that the diagnosis of obesity should be made with a BMI of ≥ 32 kg/m^2^^13^. However, this official norm does not specify the criteria for establishing this cutoff point. Recently, Aleman-Mateo et al.^10^, published the first assessment of the performance of traditional BMI cutoff points (≥ 30 kg/m^2^) for diagnosing obesity in older Latin American adults from 10 countries (n = 1154; Argentina, Brazil, Chile, Costa Rica, Guatemala, Honduras, Mexico, Peru, Paraguay, and Uruguay). They determined new BMI cutoff points for obesity of ≥ 27.5 kg/m^2^ and 26.0 kg/m^2^ for older women and older men, respectively. These cutoff points exhibited a moderate sensitivity of approximately 90% in both sexes, and moderate specificity (84.1% and 70.8% for women and men, respectively). It is important to note that this study did not report the sample size from each country. Additionally, a limitation mentioned in the study is that the sample is not representative of any of the countries. Therefore, to the best of our knowledge, this is the first study proposing newly derived BMI cutoff points for identifying obesity in older Mexican adults using a nationally representative sample and a direct marker of adiposity (FMI) in older Mexican adults.

It is important to note that BMI is the recommended indicator by the World Health Organization (WHO) for diagnosing obesity in adults aged 20 years and above, regardless of age group, ethnic group, or sex. However, previous studies have reported a low sensitivity of BMI for identifying obesity (0.42, CI: 0.31–0.43)^14^. In this regard, the new ID BMI cutoff point proposed in the present study aim to contribute to the identification of older adults with obesity in a more reliable and precise manner. According to these results, the traditional BMI cutoff point (≥ 30 kg/m^2^) has a low sensitivity for identifying obesity in older men (56.3%) and a moderate sensitivity in older women (63.1%). Sensitivity refers to the ability of BMI to correctly identify an older adult with obesity^15^. Consequently, using the traditional cutoff point leads to a significant proportion of older adults with obesity being misclassified. This underestimation of obesity prevalence is concerning as it can have adverse implications for decision-making and resource allocation in public health. In addition, the timely identification of individuals at risk in clinical settings is hindered, delaying appropriate treatment. For example, if the traditional BMI cutoff point (≥ 30 kg/m^2^ to classify obesity) is applied in our nationally representative sample, only one out of every three older adults would be classified as people with obesity. However, based on FMI, the ratio increases to one in every two older adults being diagnosed with obesity. These findings highlight the need for improved methods to accurately identify obesity in older adults, as the current approach may lead to misdiagnosis and inadequate allocation of resources.

The new ID BMI cutoff points for obesity significantly improved the ability to accurately identify individuals with obesity. In the overall sample, the new cutoff point (≥ 27.2 kg/m^2^) demonstrated high sensitivity (98.5%) and specificity (98.3%) in correctly classifying subjects with obesity, as shown in Table 3. In addition, the ratio of the proportion of individuals with obesity (based on FMI) who tested positive using the new cutoff points, compared to the proportion of normal and overweight individuals who also tested positive, was remarkably high (LR + of 56.562). Conversely, the ratio of the proportion of individuals with obesity (based on FMI) who tested negative using the new cutoff point, compared to the proportion of individuals without obesity who also tested negative, was very low for both sexes (LR− < 0.02; Table 3).

On the other hand, when evaluating the performance of the cutoff points proposed by Aleman-Mateo et al.^10^, for older Latin American adults using data from the ENSANUT 2018–2019, it is observed that sensitivity is 94.5%, and specificity of 100% in older women, in the case of older men, the sensitivity is 100%, but specificity drops to 76.5%. This leads to an overestimation of obesity prevalence in the total sample (62.0%, CI 59.6–64.4 vs. 57.7%, CI 55.3–60.1) and by sex (60.4%, CI 56.8–63.8 vs. 63.9%, CI 60.4–67.3 in older women, and 63.5%, CI 60.2–66.7 vs. 52.2%, CI 48.8–55.6 in older men) when compared to the prevalence estimated by FMI. Due to the low specificity in older men, these cutoff points classify individuals without obesity as obese. Therefore, these data suggest that the use of cutoff points of ≥ 27.5 kg/m^2^ and 26.0 kg/m^2^ for older Latin American women and older Latin American men, respectively, could have a negative impact on epidemiological studies in the Mexican population.

The performance of the new ID BMI cutoff point was assessed using data from EDLM (Older adults from northern of Mexico); Table 4, shows that the confidence intervals of prevalence of obesity was similar between the new ID BMI cutoff point and FMI. In terms of public health, this implies that the prevalence of obesity, as estimated by the new ID BMI, is comparable to that estimated by FMI. Furthermore, the sensitivity, specificity, negative predictive value, and positive predictive value of the new ID BMI cutoff point remains consistent when applied to a different sample of older adults. By adopting these new BMI cutoff point, public health initiatives can make more informed decisions and allocate resources more effectively. Moreover, in the clinical setting, healthcare professionals can promptly identify individuals at risk and provide appropriate interventions. This significant improvement in the identification of obesity in Hispanic older adults has far-reaching implications, as it contributes to better healthcare planning and management in addressing this prevalent health concern.

The main limitation of this study is that fat mass was estimated through a predictive equation that despite being developed using the four-compartment method and being validated in Hispanic older adults, is a double indirect method for estimating fat mass. However, despite these limitations, the results obtained from the “external validation” by a sub-analysis of the EDLM dataset, showed consistent findings with those obtained from the ENSANUT 2018–2019 database. This consistency highlights the improved sensitivity and accuracy achieved by the new ID BMI cutoff point in correctly classifying subjects with obesity. It is noteworthy that the EDLM dataset, although not a representative sample, included data from 1055 subjects, providing a substantial sample size for analysis. Therefore, despite the limitations associated with the estimation of fat mass, the high performance of the new ID BMI cutoff points observed in both datasets is noteworthy.

## Conclusions

The current BMI cutoff point (≥ 30 kg/m^2^) is unreliable and underestimates the prevalence of obesity in older Hispanic adults. However, the newly proposed BMI cutoff point (≥ 27.2 kg/m^2^ for both older men and older women) substantially improve the ability to identify older individuals with obesity. This enhanced identification of obesity allows for a more accurate estimation of its prevalence and enables timely intervention and treatment in this specific age group.

## Data Availability

The datasets generated during and/or analyzed during the current study are available from the corresponding authors on reasonable request.
